# IoTSim: Internet of Things-Oriented Binary Code Similarity Detection with Multiple Block Relations

**DOI:** 10.3390/s23187789

**Published:** 2023-09-11

**Authors:** Zhenhao Luo, Pengfei Wang, Wei Xie, Xu Zhou, Baosheng Wang

**Affiliations:** College of Computer, National University of Defense Technology, Changsha 410073, China

**Keywords:** IoT security, binary code similarity detection, vulnerability detection

## Abstract

Binary code similarity detection (BCSD) plays a crucial role in various computer security applications, including vulnerability detection, malware detection, and software component analysis. With the development of the Internet of Things (IoT), there are many binaries from different instruction architecture sets, which require BCSD approaches robust against different architectures. In this study, we propose a novel IoT-oriented binary code similarity detection approach. Our approach leverages a customized transformer-based language model with disentangled attention to capture relative position information. To mitigate out-of-vocabulary (OOV) challenges in the language model, we introduce a base-token prediction pre-training task aimed at capturing basic semantics for unseen tokens. During function embedding generation, we integrate directed jumps, data dependency, and address adjacency to capture multiple block relations. We then assign different weights to different relations and use multi-layer Graph Convolutional Networks (GCN) to generate function embeddings. We implemented the prototype of IoTSim. Our experimental results show that our proposed block relation matrix improves IoTSim with large margins. With a pool size of $10^{3}$, IoTSim achieves a recall@1 of 0.903 across architectures, outperforming the state-of-the-art approaches Trex, SAFE, and PalmTree.

## 1. Introduction

Nowadays, the wide usage of Internet of Things (IoT) devices in various fields, including smart medical care and smart homes, has significantly improved people’s lives. According to a report by IoT statistics [1], the number of connected IoT devices is expected to exceed 29 billion by 2030. However, the rapid growth in demand for IoT devices has led to the development of IoT firmware heavily relying on third-party components (TPCs), often without necessary security audits. Although this way improves development efficiency and reduces costs, it also exposes the firmware to vulnerabilities and weaknesses, making them attractive targets for attackers. Numerous security issues [2,3,4,5,6,7] indicate the fragility of the current IoT ecosystem, raising public concern about IoT security risks.

However, detecting these vulnerabilities in IoT firmware is challenging due to the following reasons. Firstly, many IoT firmware images only provide binary files, making source code unavailable for security analysis. Secondly, IoT firmware originates from different instruction set architectures (ISAs), necessitating extensive reverse engineering expertise and specialized knowledge for security analysis. Thirdly, analyzing more than 29 billion IoT devices and discovering their vulnerabilities significantly burdens researchers. Therefore, there is an urgent need for an accurate and automated technique to identify these vulnerable TPCs and vulnerable functions. As a result, binary code similarity detection (BCSD) has become an active research focus for detecting vulnerabilities hidden in IoT devices.

**Figure 1:**
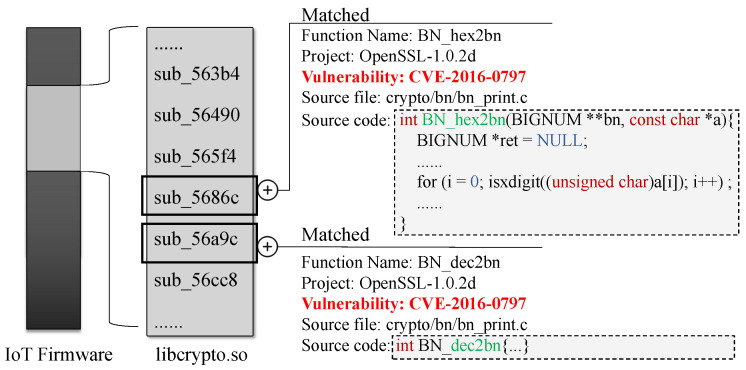
An example of using BCSD in IoT firmware analysis.

Binary code similarity detection is a fundamental technique in computer security that can detect similarities between two binary code snippets. It is widely used for various applications, including vulnerability detection [8,9,10,11,12,13,14,15,16,17,18,19,20,21,22], malware analysis [23,24,25,26,27,28], and binary patch analysis [29,30,31]. Figure 1 shows an example of BCSD usage in firmware security analysis. Given an IoT firmware without symbol tables, the BCSD approaches extract its functions and match them with functions in the vulnerability database based on function similarity to detect vulnerable functions in the firmware. For example, function sub_5686c is matched with function BN_hex2bn, which is associated with the vulnerability in the Common Vulnerabilities and Exposures (CVE) database. Furthermore, according to the BCSD results, the function name, the symbol table, the project name, and even the source code can be restored. This can locate vulnerabilities in massive IoT firmware and provide reverse analysts with critical information, e.g., source code and symbol tables.

Prior to the utilization of machine learning (ML) in Binary Code Similarity Detection (BCSD) tasks, traditional BCSD approaches [20,21,32,33] heavily rely on specific features, including control flow graphs (CFGs), the count of basic blocks, and string constants. However, determining the weights of these syntactic features requires extensive experience and expert knowledge, and they may vary with different compilers and optimization options. Moreover, traditional graph-based methods such as graph isomorphism matching are excessively time-consuming for analyzing large-scale firmware, leading to relatively lower accuracy and scalability.

In recent years, researchers have increasingly adopted learning-based approaches to tackle BCSD tasks, and the current state-of-the-art BCSD approaches [11,13,14,34,35] are predominantly based on machine learning (ML) techniques. These approaches typically involve the disassembly of binary code into either assembly language or intermediate representation (IR). Subsequently, trained neural networks are utilized to extract the semantic information from functions and embed them into high-dimensional representations. Finally, function matching is performed based on the similarity of these embeddings. Gemini [36] manually selects statistical features of basic blocks and employs graph neural networks (GNNs) to generate function embeddings for function matching. More recently, the field of natural language processing (NLP) has achieved significant advancements in semantic extraction. Consequently, NLP techniques have been introduced into BCSD tasks by methods such as jTrans [11], PalmTree [34], and SAFE [35]. These techniques aim to automatically extract semantics and generate function embeddings for calculating function similarity. Despite the progress made by learning-based models, they still have limitations when it comes to new application scenarios of the BCSD for IoT firmware.

**Figure 2:**
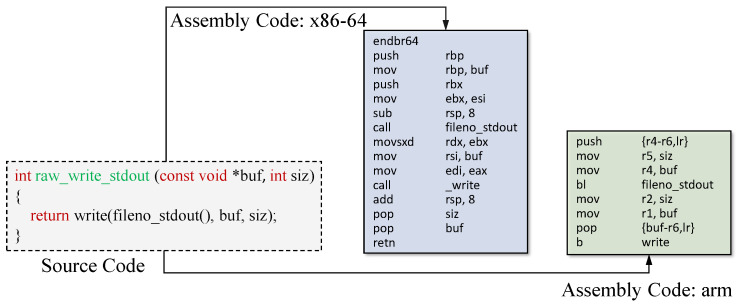
Assembly code on the different architectures. Both assembly sequences are from the same source code in the OpenSSL project. However, their opcodes, operands, and calling conventions are very different due to the different architectures.

**P1**: IoT firmware originates from different architectures such as x86, arm, and mips. This leads to the inclusion of instructions from different architectures, and poses significant risks of encountering out-of-vocabulary (OOV) challenges. OOV problems are widely recognized in the NLP field. When a word has not been encountered during training, it is referred to as an OOV word, and the word embedding model becomes incapable of generating semantic representations for such words. The OOV issue in BCSD for IoT firmware is further exacerbated by the existence of various instruction sets, address offsets, and registers. Figure 2 illustrates the assembly code compiled from the same source code but targeting different architectures (i.e., x86-64 and arm). Notably, the assembly code generated for these two architectures exhibits stark dissimilarities. As a consequence, considerable differences arise at the lexical and syntactic levels, resulting in severe OOV challenges.

For OOV words, existing approaches [11,34,35,37] use normalization based on pre-defined rules to deal with string literals, immediate numbers, and address offsets. However, when these special words are replaced with dummy tokens, the essential semantics may be compromised. Moreover, when confronted with unknown expressions found in IoT firmware, these approaches still encounter OOV challenges. For example, despite being trained on multiple ISAs, SAFE [35] still suffers from OOV issues and does not exhibit satisfactory performance in cross-architecture BCSD tasks.

**P2**: Existing approaches extract superficial features from Control Flow Graphs (CFGs), such as adjacency matrices, which overlooks critical semantic information. A CFG represents all possible paths during execution, which consists of basic blocks. Multiple relations exist between connected basic blocks: (1) edges between basic blocks are directed, (2) address adjacent basic blocks often have a stronger mutual influence, and (3) data flow dependencies may exist between basic blocks. Adjacency matrices alone cannot capture these in-depth multiple relations. Neglecting these semantic and structural nuances makes it challenging to build an accurate model for BCSD tasks for IoT firmware. Previous approaches [19,36] primarily concentrate on connections between basic blocks, disregarding various associations like data dependencies, which results in low performance.

To solve the aforementioned problems, in this paper, we present IoTSim, a novel deeply customized cross-architecture approach for binary code similarity detection in IoT firmware, which supports vulnerability discovery and firmware component analysis. We use an NLP-based model DeBERTa [38] to capture basic block semantics, and use GNNs to capture the control and data flow information, to generate binary function semantic embeddings. To resolve the challenges in problem **P1**, we lift assembly code into an intermediate representation, namely microcode, to mitigate differences in instruction sets, registers, and calling conventions. During the training phase, we do not use normalization to deal with string literals and immediate numbers, in order to preserve important semantics. For OOV issues in testing, we tokenize these OOV words into their base-tokens and generate semantic embeddings. To this end, we propose a newly designed pre-training task, namely Base-Token Prediction (BTP), so that base-tokens include the basic semantics of their types. Furthermore, in binary code, the attention weight of an instruction pair depends on not only their contextual instructions but also their relative positions. Adjacent instructions tend to have stronger dependencies. Therefore, we utilize relative position embeddings based on disentangled attentions to capture content-to-content (c2c), content-to-position (c2p), and position-to-content (p2c) attentions for higher precision.

To address problem **P2**, we propose using directed adjacency, data dependency, and address matrices to represent relations between basic blocks. For multiple relations between connected basic blocks, first, we determine the direction relations based on the predecessors and successors of the basic block. Then, according to the address and connection of the basic blocks, the larger address distances between blocks generally indicate weaker mutual influence. Furthermore, variables in binary code are required to be defined before being used. Based on the def-use chains at the basic block level, we can determine the data dependencies between basic blocks. Thus, we generate an abundant data-based control flow graph (DCFG) to represent the CFG deeply. Section 5.3 shows that DCFGs can improve recall@1 by 15.5±6.1% compared to CFGs solely in the function level BCSD tasks with $10^{3}$ candidates.

In summary, we have made the following contributions:

- We propose a novel deeply cross-architecture approach using NLP techniques for IoT-oriented binary code similarity detection tasks. To resolve problem **P1**, we lift assembly code into microcode and propose a newly designed pre-training task to mitigate OOV issues;
- To resolve problem **P2**, we consider multiple relations between basic blocks to generate DCFGs to capture rich contextual information between basic blocks. We then use a GNN model to integrate basic block embeddings based on DCFGs for generating function embeddings;
- We implement IoTSim which can be used for vulnerability detection and firmware component analysis in the real world. We evaluate IoTSim with extensive experiments. The experiments show that IoTSim outperforms the state-of-the-art approaches such as Trex, SAFE, GMN, and PalmTree.

The remaining sections of the paper are structured as follows: Section 2 provides an overview of the relevant literature. Section 3 clarifies key points of this paper. In Section 4, we present a detailed description of the design of IoTSim. Section 5 conducts extensive experiments to evaluate the performance of IoTSim. Lastly, Section 6 concludes the paper and provides final remarks.

## 2. Related Work

In this section, we provide a concise overview of the relevant literature concerning binary code similarity detection. We discuss both mono-architecture approaches and cross-architecture approaches.

**Mono-architecture Approaches**: Extensive progress has been made in research on detecting binary code similarity for mono-architecture binaries. For example, various approaches [20,23,28,39,40] utilize syntax, structural, and statistical features to match similar binary functions. Tracelet [41] decomposes binary functions into continuous traces and measures the similarity between two traces by constraint solving and data dependencies. BLEX [42] executes functions under a randomized environment and compares their similarity based on the corresponding I/O values collected. BinSim [23] proposes a hybrid fine-grained approach using system call sliced segment to identify binary code similarities with symbolic execution. Similarly, CoP [43] employs symbolic execution and a theorem prover to compare binary code similarity by matching the longest common sub-sequence with basic blocks as elements. Nonetheless, due to their computational complexity, these approaches may not be practical when dealing with extensive function repositories. Drawing inspiration from Natural Language Processing (NLP) techniques, many researchers [11,14,22,34,44,45] introduce language models to extract the semantics of binary code for BCSD tasks. Ding et al. [14] propose Asm2Vec, which represents binary functions as high-dimensional embeddings and utilizes the Distributed Memory Model of Paragraph Vectors (PV-DM) model [46] to extract semantics from binary functions for function embedding generation. Li et al. [34] employ PalmTree, a transformer-based NLP model using BERT [47], to measure the similarity of binary code in assembly language. Additionally, jTrans [11] presents a transformer-based approach that incorporates control flow information through jump-aware representation. These approaches involve representing binary functions as high-dimensional embeddings, facilitating the search for similar candidate functions within extensive function repositories. However, in the IoT scenarios, firmware images and executable files from different architectures require BCSD approaches to support finding similar functions across various architectures.

**Cross-architecture Approaches**: With the urgent need for cross-architectural BCSD approaches, recent research has focused on cross-architecture BCSD tasks. Traditional methods typically involve selecting architecture-robust features, such as statistical, syntactic, and structural features, to compute the similarity of binary code. These include BinDiff [32], DiscovRE [48], Esh [15], GitZ [49], Genius [19], and Gemini [36]. Esh [15] and GitZ [49] decompose binary procedures into comparable fragments using data flow analysis and use a statistical framework to detect similar binary fragments. Genius [19] and Gemini [36] adopt machine learning and consider statistical features as attributes of CFGs to graph embeddings for BCSD tasks. Both of them rely on hand-crafted features, which necessitate rich experience and domain knowledge to match similar functions. Trex [13] proposes a transformer-based model using micro-traces to capture execution semantics of functions for cross-architecture BCSD tasks. VulHawk [50] integrates basic block features and CFGs to detect vulnerabilities across architectures. SAFE [35] trains a Word2Vec model [51] using binaries from various architectures, including x86 and arm, to detect binary code across different architectures. However, in our experiments, we observe that despite being trained using multiple architecture binaries, SAFE still suffers from a large number of OOV issues in the experiments, which severely affects its performance.

Furthermore, there are multiple relations (e.g., directed jumps, data dependency, and address adjacency) in binary functions, which contain important semantic information for BCSD tasks. The existing approaches use single basic block relations and do not deeply consider the combination of multiple relations, which may miss critical semantic information to distinguish dissimilar functions with minor differences. Facing high pool size scenarios, it is difficult for them to achieve good performance in numerous candidate functions.

## 3. Problem Definition

In this section, we aim to clarify key points of this paper to enhance the clarity of the presentation.

**Detection Granularity**. This paper focuses on measuring the similarity of two binaries at the function level. In the analysis of IoT firmware, giving the similarity and symbol tables of functions can help researchers understand binary code and greatly reduce the manual workload.

**Similarity Explanation**. In the existing BCSD literature [14,27,52], there are four types of function similarity: (1) literal identity, (2) syntactic equivalence, (3) functional equivalence, and (4) the same or logic similar source code. Due to the usage of different compilers and optimization options in IoT firmware, there may still be literal and syntactic differences among binary functions originating from the same source code. The same functionality has various implementations (e.g., bubble sort and quick sort), but they cannot share the symbol tables. Consequently, types (1), (2) and (3) are not suitable for vulnerability discovery and firmware component analysis with symbol table supplementation. Our focus lies on type (4), which involves binary functions that may exhibit syntactic differences but share similar functional logic in their source code.

**Binary Code Similarity Detection**. BCSD approaches are employed to calculate the similarity between two binary functions. In IoT firmware analysis, binary functions are compiled using diverse compilers (e.g., GCC and Clang) with various optimization options (e.g., O0, O1, O2, O3, and Os) on multiple architectures (e.g., arm, mips, and x86). As a result, even when two functions originate from the same source code, they may have different instructions and structures. Therefore, an effective IoT-oriented BCSD approach should be robust across architectures, compilers, and optimization options. Furthermore, it also supports working on large pools of candidate binary functions. In practice, given a query function, BCSD approaches need to calculate the similarity between the query function and a large pool of candidate functions, selecting the most similar one [11]. This requires accurately identifying the most similar functions from the candidate pool while distinguishing irrelevant ones.

## 4. Design

**Figure 3:**
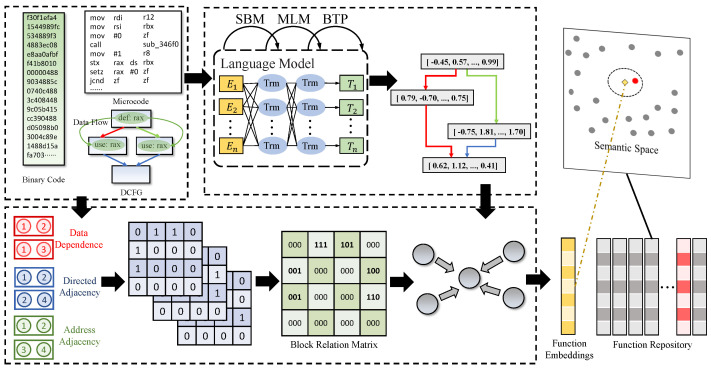
The overview of IoTSim, which consists of a preprocessor, a block semantic model, and a function embedding model.

To address the problems mentioned in Section 1, we propose a BCSD approach named IoTSim, for IoT-oriented BCSD tasks. Figure 3 shows the overview of IoTSim, including three modules to implement its functionality.

**Preprocessor**. Firstly, IoT binaries are disassembled and their binary code is transformed into microcode. By analyzing the def-use chains, we obtain data dependencies based on defined variables and used variables for each basic block. Subsequently, CFGs and data dependencies are integrated to generate data-based control flow graphs (DCFGs). Based on the various relations between basic blocks in DCFGs, we create the address matrix, the directed matrix, and the data dependencies.

**Block Semantic Model**. This module utilizes an NLP model to produce semantic embeddings for basic blocks using microcode sequences. We employ a language model based on DeBERTa [38] to construct basic block embeddings. To optimize model parameters, we utilize two pre-training tasks: (1) the masked language model (MLM) task helps the model learn semantic relations between microcode; and (2) the base-token prediction (BTP) task assists the model in learning and complementing the semantics of base-tokens.

**Function Embedding Model**. This module combines basic block embeddings and graph features to generate function embeddings. We establish a block relation matrix that incorporates multiple relations such as directed adjacency, data dependencies, and address adjacency. Leveraging the block relation matrix and basic block embeddings, we utilize graph neural networks (GNNs) to capture control-flow and data-flow relations for generating function embeddings. During training, we use the normalized temperature-scaled cross-entropy loss (NT-Xent) [53] to optimize the model parameters, making the similar functions’ embeddings closer in the semantic space.

### 4.1. Preprocessor

The preprocessor generates the function features used by the Block Semantic Model and Function Embedding Model, including instructions, control-flow, and data-flow features.

**Figure 4:**
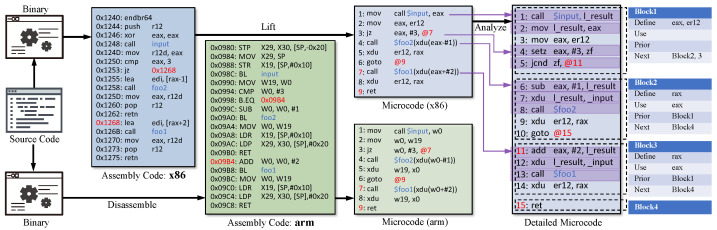
An example control flow of a binary function. The left is the linear layout assembly code with jump addresses, and the right is the corresponding control-flow graph.

We initially disassemble binary files and convert their binary code into microcode to address the cross-architecture differences. The microcode is an architecture-agnostic intermediate representation from IDA Pro [54]. Figure 4 illustrates the conversion of assembly code from diverse architectures into microcode. Notably, there are significant dissimilarities in assembly snippets when comparing the same source code across different architectures (e.g., x86 and arm). Their registers (e.g., eax and W0), opcodes (e.g., jz and B.EQ), and calling conventions look completely different. After converting to microcode, they have the same opcodes and calling conventions. Consequently, this conversion mitigates the differences introduced by instruction sets and calling conventions. Within the microcode, intricate instruction nests can be observed, as demonstrated in Ln. 7 of the microcode (x86) in Figure 4, which are compounded by multiple semantics and are susceptible to OOV issues. To address such OOV problems, we analyze sequences of microcode instructions and split these nests into individual instructions. We consider an instruction with multiple operands nested within it to be an instruction nest. For example, the instruction of Ln.7 in microcode (x86) is an instruction nest, which nests three opcodes call, xdu, and add. After analysis, we split it into three sub-instructions of Ln.11-13 in detailed microcode. These instructions are semantically equivalent to the original instruction nest and can reduce the OOV issues caused by instruction nesting.

To capture the data dependency among basic blocks, we use def-use chains to capture defined variables and used variables for each basic block to generate Data-based Control Flow Graphs (DCFGs). In binary code, variables should be defined before being used. The def-use chains enable us to monitor the usage and definition of variables. Here, we focus on defining and using variables at the basic block level. For example, in optimized microcode, Block3 uses eax, which is defined by Block1. This indicates a data dependency between Block1 and Block3. We integrate CFGs based on prior and next blocks of basic blocks, and data dependencies based on the definition and use of variables between basic blocks, to construct DCFGs for binary functions.

### 4.2. Block Semantic Model

In this section, we employ a customized transformer-based language model with disentangled attention to learn microcode semantics and generate semantic embeddings at the basic block level. Compared with instructions, basic blocks have richer semantics; and compared with functions, basic blocks do not have complex multi-branch structures, which facilitates semantic extraction. We consider instructions as words and basic blocks as sentences. Considering the important influence of instruction content and position on block semantics, we adopt disentangled attention to capturing relative position semantics. Subsequently, we utilize two self-supervised pre-training tasks, namely Masked Language Model (MLM) and Base-Token Prediction (BTP), to train our model.

#### 4.2.1. Language Model

**Figure 5:**
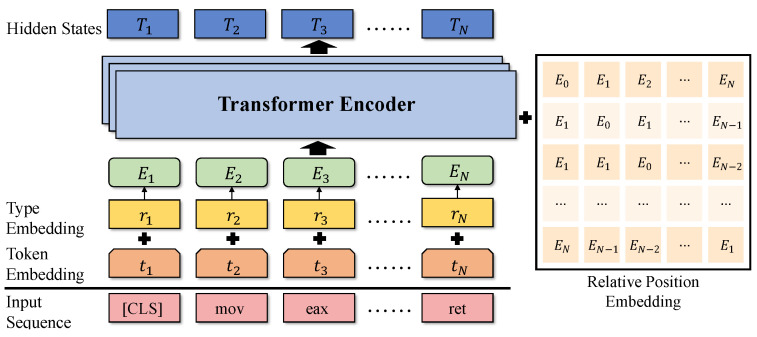
The architecture of the language model.

The transformer-based architectures have shown encouraging results in NLP tasks. Our model is built upon the DeBERTa [38] model, one of the state-of-the-art NLP models. Figure 5 illustrates the architecture of our language model, comprising stacked transformer blocks for generating embeddings. For each input sequence, we utilize a tokenizer to convert microcode into token objects. These tokens are then processed through transformer stacks to produce hidden states, with the final hidden states representing the semantics of the microcode.

**Tokenizer.** The problem of out-of-vocabulary (OOV) is widely recognized in NLP. In the context of BCSD for IoT firmware, the OOV issue is further amplified due to the presence of various instruction sets. Even when converting binary code into microcode, the OOV issue can still arise from string literals, address offsets, registers, and function names. To mitigate the OOV issues, Inter-BIN [55] adopts a character-level tokenizer, which has a smaller vocabulary and rarely suffers from OOV issues. However, a single character is less meaningful and may lead to incorrect semantics. Also, it requires more computing resources for each character computation. And, other approaches [11,35,37] replace special words (e.g., string literals and constant values) with dummy tokens, which may lose important semantics.

We perform tokenization of the microcode at the opcode/operand level. To handle low-frequency operands, we replace them with their base-tokens instead of using dummy tokens. The microcode provides a basic type for each operand. For example, the addresses 0x400C8D and 0x400A30 are both specific address offsets that may result in out-of-vocabulary (OOV) issues, while microcode categorizes them into the address type (mop_a), which represents their common basic semantics. This is particularly useful for addressing OOV issues, as it allows us to map OOV words to their corresponding base-tokens based on their basic types. To construct our vocabulary, we initially iterate through all the microcode and filter out infrequently occurring tokens with frequencies lower than 100. Subsequently, we include all basic types in our vocabulary as base-tokens. During the tokenization process, any OOV words are mapped to their corresponding base-tokens according to their basic types.

**Relative Position Embedding.** The positions of instructions play a crucial role in determining basic block semantics. Recent studies [38,47,56,57] have shown that relative positions are more effective than absolute positions for NLP tasks. Inspired by DeBERTa [38], in each transformer encoder layer, we utilize disentangled attentions in each transformer encoder layer to generate relative position embeddings that capture content-to-content (c2c), content-to-position (c2p), and position-to-content (p2c) attentions. The cross attention score between tokens *i* and *j* is calculated as follows:

(1) $$\begin{array}{cc} A_{i,j} & =\left\{H_{i},P_{i|j}\right\}\times {\left\{H_{j},P_{j|i}\right\}}^{⊺}\\ \; & =\underset{c2c}{\underset{︸}{H_{i}H_{j}^{⊺}}}+\underset{c2p}{\underset{︸}{H_{i}P_{j|i}^{⊺}}}+\underset{p2c}{\underset{︸}{P_{i|j}H_{j}^{⊺}}}+\underset{p2p}{\underset{︸}{P_{i|j}P_{j|i}^{⊺}}} \end{array}$$

where $H_{i}$ and $P_{i|j}$ represent its content and relative position with the token *j*, respectively. In microcode, the position-to-position (p2p) term does not provide much additional information, we do not consider it in implementation.

The formulation for the standard self-attention calculation [58] is as follows:

(2) $$\begin{array}{cc} Q={\mathrm{HW}}_{q},\;K & ={\mathrm{HW}}_{k},\;V={\mathrm{HW}}_{v}\\ H_{o} & =\mathrm{softmax}(\frac{A}{\sqrt{d}})V \end{array}$$

where $H\in R^{N\times d}$ is the input hidden vectors, $H_{o}\in R^{N\times d}$ represents the output of self-attentions, Q, K, and V are three matrices, and $W_{q}$, $W_{k}$, and $W_{v}$ are their projection matrices. We put the content input H and the relative position P into Equation (2), where $W^{c}$ and $W^{r}$ represent content position matrix and relative position projection matrix, respectively.

(3) $$\begin{array}{cc} \; & Q_{c}={\mathrm{HW}}_{q}^{c},\;K_{c}={\mathrm{HW}}_{k}^{c},\;V={\mathrm{HW}}_{v}^{c},\\ \; & Q_{r}={\mathrm{PW}}_{q}^{r},\;K_{r}={\mathrm{PW}}_{k}^{r} \end{array}$$

According to Equations (1) and (3), the elements ${\tilde{A}}_{i,j}$ of attention matrix $\tilde{A}$ are calculated as follows:

(4) $$\begin{array}{cc} \; & {\tilde{A}}_{i,j}=Q_{i}^{c}K_{j}^{c}⊺+Q_{i}^{c}K_{\delta (i,j)}^{r}⊺+K_{j}^{c}Q_{\delta (j,i)}^{r}⊺ \end{array}$$

where δ(i,j) represent the maximum relative distance from token *i* to token *j*, and the output $H_{o}$ of disentangled attentions can be formulated as:

(5) $$H_{o}=\mathrm{softmax}\left(\frac{\tilde{A}}{\sqrt{3d}}\right)V_{c}$$

We feed the output $H_{o}$ into a fully connected feed-forward network to obtain the transformer encoder layer output.

#### 4.2.2. Pre-Training Tasks

For large-scale training our model, we incorporate the Masked Language Model (MLM) task, similar to other NLP model training approaches, but with domain-specific adaptations. Specifically, we propose a novel pre-training task, Base-Token Prediction (BTP), to improve the semantic understanding of base-tokens in IoTSim.

**Masked Language Model.** This model is employed to perform fill-in-the-blank tasks, enabling the model to utilize the tokens surrounding a mask token for predicting the masked token [47]. Through the MLM task, the model learns the connections between tokens.

Given a token sequence $X=\{x_{i}|i\in \left(0,n\right)\}$, we randomly select 15% of the token sequence to be replaced. Among the selected tokens, 80% of them are substituted by the [MASK] token, 10% are replaced with other tokens, and 10% remain unchanged. This replacement process yields a masked sequence denoted as $\tilde{X}$. Subsequently, we input $\tilde{X}$ into the block semantic model and feed the output into an MLM head to reconstruct X by predicting the masked tokens $\tilde{x}$ conditioned on $\tilde{X}$. The loss function is as follows:

(6) $$L_{MLM}\left(\theta_{1},\theta_{2}\right)=-\log p_{\left\{\theta_{1},\theta_{2}\right\}}\left(X|\tilde{X}\right)$$

where $\theta_{1}$ and $\theta_{2}$ are the parameters of the block semantic model and the MLM head, respectively.

**Base-Token Prediction.** In our model, the tokenizer is utilized to replace OOV operands with their base-tokens. To establish semantic connections between tokens and their respective base-tokens, we propose a task called Base-Token Prediction (BTP). This task aims to facilitate the learning of common semantics among basic types of tokens by their corresponding base-tokens.

**Figure 6:**
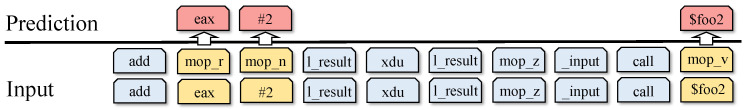
Base-token prediction.

Given a sequence of tokens denoted as $X=\{x_{i}|i\in \left(0,n\right)\}$, we randomly select 15% of the token sequence to substitute them with their base-tokens. As a result, we obtain a replaced sequence denoted as ${\tilde{X}}_{b}$. Figure 6 illustrates an example of the BTP task, where tokens such as eax (register), #2 (immediate number), and $foo2 (function name) are selected. These tokens are replaced with their respective base-tokens; for instance, #2 is substituted with mop_n, which represents immediate number constants.

We feed ${\tilde{X}}_{b}$ into the block semantic model and feed the output into a BTP head to reconstruct X by predicting the replaced tokens $\tilde{x}$ conditioned on $\tilde{X}$. The loss function is as follows:

(7) $$L_{BTP}\left(\theta_{1},\theta_{3}\right)=-\log p_{\left\{\theta_{1},\theta_{3}\right\}}\left(X|\tilde{X}\right)$$

where $\theta_{1}$ and $\theta_{3}$ are the parameters of the block semantic model and the BTP head, respectively.

The loss function of the block semantic model is the combination of loss functions:

(8) $$L=L_{MLM}+L_{BTP}$$

### 4.3. Function Embedding Model

The objective of the function embedding model is to generate semantic function embeddings by integrating basic block embeddings and graph features. Binary functions encompass both control-flow information and data-flow information. Control-flow information describes the potential execution paths of a function, while data-flow information describes the data dependencies between basic blocks. Hence, these semantic and structural features are crucial in the generation of function embeddings.

We first generate directed matrices, data dependency matrices, and address matrices according to DCFGs. Then, we combine these matrices into a block relation matrix. Finally, we integrate the block relation matrix and block embeddings to generate function embeddings using Graph Convolutional Networks (GCNs) [59].

#### 4.3.1. Block Relation Matrix

In binary functions, basic blocks have multiple relations. First, connected basic blocks are directed. Second, basic blocks with adjacent addresses indicate relatively more dependencies. Third, data flow dependencies exist between connected basic blocks or not. These relations result in different mutual influences between basic blocks. Capturing these multiple relations is critical to building an accurate function embedding model for BCSD tasks.

**Figure 7:**
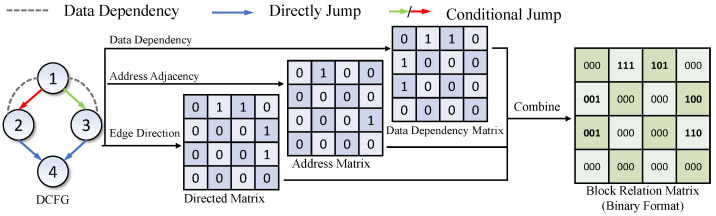
Block relation matrix.

Figure 7 shows an example of constructing a block relation matrix. In the Figure, the DCFG is composed of 4 basic blocks, and there are different relations between these basic blocks. For example, ➀ Block1 and Block2 are adjacent and have data dependencies, ➁ Block2 and Block4 have a directed edge, and ➂ Block2 and Block3 have no relations. We first base on directed edges, data dependencies, and address adjacency of the DCFG to generate its directed matrix, data dependency matrix, and address matrix, respectively. Then, we combine the above three matrices to construct the block relation matrix. The combination formula is as follows:

$$m_{B}=\left(m_{A}<<2\right)+\left(m_{C}<<1\right)+m_{D}$$

where $m_{B}$, $m_{A}$, $m_{C}$, and $m_{D}$ represent the corresponding elements in the block relation matrix, directed matrix, address matrix, and data dependency matrix, respectively, and << denotes the left shift operation. The constructed block relation matrix distinguishes different relations between basic blocks. For example, the relation from Block1 to Block2 is represented as 111, the relation from Block2 to Block4 is represented as 100, and the relation from Block2 to Block3 is represented as 000, which indicates no relations.

#### 4.3.2. Function Embedding Generation

A binary function consists of basic blocks that exhibit mutual influences. In order to generate function embeddings, we combine the basic block embeddings produced by the Block Semantic Model with the block relation matrix. To deeply combine graph information and basic block embeddings, we utilize Graph Convolutional Networks (GCNs) [59] for embedding generation. Within this framework, we view binary functions as attributed graphs, where the basic blocks serve as nodes and their corresponding embeddings serve as node attributes. The block relation matrix indicates different relations between nodes in the graph. We feed basic block embeddings and the block relation matrix into a multi-layer GCN. $X^{\left(\ell \right)}$ represents the node embeddings of the *ℓ*-th layer, and B represents the block relation matrix. The *ℓ*-th layer GCN’s output $X^{\left(\ell +1\right)}$ is computed as follows:

(9) $$\begin{array}{cc} \; & A=F(B)\\ \; & \tilde{A}=A+I_{N}\\ \; & {\tilde{D}}_{ii}=\sum {\tilde{A}}_{ij}\\ \; & X^{\left(\ell +1\right)}=\sigma \left({\tilde{D}}^{-\frac{1}{2}}\tilde{A}{\tilde{D}}^{-\frac{1}{2}}X^{\left(\ell \right)}W^{\left(\ell \right)}\right) \end{array}$$

where A is a weighted adjacency matrix which encodes the relations of B. Here, we use a learnable function F(B) to encode different relation types into numeric weights. $\tilde{A}$ represents a weighted adjacency matrix that includes self-connections. $I_{N}$ is an identity matrix. ${\tilde{D}}_{ii}$ denotes the degree matrix of each node, while $W^{\left(\ell \right)}$ represents a trainable weight matrix specific to each layer. The activation function σ is the rectified linear unit ReLU(·). To comprehensively learn the semantic and structural aspects of CFGs, we adopt a 16-layer GCN to propagate block semantics using the block relation matrix. Finally, by applying mean pooling on the outputs of the final GCN layer, we generate function embeddings.

#### 4.3.3. Model Training

In the BCSD tasks, the main objective of the Function Embedding Model is to map similar functions to nearby regions in the embedded space. To measure the similarity between functions, we utilize the cosine similarity score of function embeddings. By leveraging Debugging with Attributed Record Formats (DWARF) information, it becomes feasible to automatically generate ground truth for function similarity based on function names and source files. Therefore, supervised training is employed to optimize the Function Embedding Model.

**Function Ground truth.** We automatically construct a function ground truth based on function names and the source files. Given a function *f*, we pick the functions whose names are the same as *f* from the same project, and label them as a similar function group $F_{sim}$. We randomly sample the functions whose names differ from *f*, and label them as a similar function group $F_{dissim}$ against *f*. These functions of $F_{sim}$ and $F_{dissim}$ can be compiled by different compilers (e.g., GCC and Clang) with different optimization levels (O0, O1, O2, O3, and Os) on any architectures (e.g, x86, arm, and mips), which increases the diversity of the dataset.

**Training Objective.** During the training process, we aim to maximize the cosine similarity scores between embeddings of similar function pairs while minimizing those between embeddings of dissimilar function pairs. The number of dissimilar function pairs in the real world is much greater than that of similar function pairs. Given a binary function $f_{i}$, we pick one of the corresponding similar functions $f_{j}$ from the dataset $D_{F}$ based on its function name and source code. The function $f_{j}$ can come from different architectures, compilers, and optimization levels against function $f_{i}$. The Function Embedding Model encodes both $f_{i}$ and $f_{j}$ to generate the function embeddings $e_{i}$ and $e_{j}$. We randomly sample N−1 functions from $D_{F}$ to construct negative samples against the function $f_{i}$. We use the normalized temperature-scaled cross-entropy loss (NT-Xent) [53] as the training loss:

(10) $$L_{Function}=-\log\frac{\exp(\cos\left(e_{i},e_{j}\right)/\tau )}{\sum_{k=1}^{N}1_{\left[k\neq i\right]}\exp\left(\cos\left(e_{i},e_{k}\right)/\tau \right)}$$

where cos(·) denotes the cosine similarity score function, 1 is the indicator, and τ is a hyper-parameter which controls the temperature.

## 5. Evaluation

In this section, we evaluate IoTSim and answer the following research questions:

- RQ.1: can IoTSim effectively identify similar function pairs when given functions from different compilers, architectures, and optimization levels?
- RQ.2: how much does DCFG contribute to the performance of IoTSim?
- RQ.3: what are the applications of IoTSim in practice?

### 5.1. Implementation and Setup

We utilized Python v3.8.5 and PyTorch [60] to implement the IoTSim framework. We employed the DeBERTa model based on Transformers [61] and Graph Convolutional Networks (GCNs) relying on PyTorch Geometric [62]. By default, our DeBERTa model consists of six layers, and the output embeddings are set to a dimension of 256. In the Function Embedding Model, we employ 16-layer GCNs for generating embeddings, and the NT-Xent loss function adopts a hyper-parameter τ with a value of 0.1. The model training and evaluation experiments were conducted on a desktop computer operating Windows 10, equipped with an Intel Core i9-10920X CPU, 128 GB RAM, and one NVIDIA RTX 3090 GPU. The model training process lasted for one week, during which we retained the best-performing checkpoints for evaluation.

#### 5.1.1. Baselines

To provide a comprehensive comparison, we select the following state-of-the-art approaches as baselines for evaluation. The chosen baseline approaches are as follows:

- Graph Matching Networks (GMN). Marcelli et al. [63] show that a GMN based on CFGs has natural advantages in cross-architecture scenarios;
- PalmTree [34], one of the state-of-the-art BCSD methods, employs pre-trained models using the BERT model to generate semantic embeddings for binary code (https://github.com/palmtreemodel/PalmTree, accessed on 10 March 2023);
- SAFE [35] uses a word2vec model [51] and a recurrent neural network to generate function embeddings (https://github.com/facebookresearch/SAFEtorch, accessed on 10 March 2023);
- Trex [13], the state-of-the-art BCSD approach, uses transfer-learning-based models that utilize micro-traces to generate function embeddings for comparing similar functions (https://github.com/CUMLSec/trex, accessed on 10 March 2023).

These selected baselines are representative BCSD approaches involving NLP techniques, CFG, and micro-traces. By comparing IoTSim with these state-of-the-art approaches, we aim to conduct a comprehensive evaluation of its performance improvements.

#### 5.1.2. Benchmarks

To evaluate IoTSim in depth and detail, we used the following two datasets:

Dataset-1 is a function dataset, which is used to evaluate the performance of IoTSim at the function level. We construct Dataset-1 using including seven projects, i.e., Linux-source, CoreUtils, OpenSSL, DiffUtils, FindUtils, Libmicrohttpd, and SQLite. These projects are compiled using two compilers (GCC and Clang) with five optimization levels (O0, O1, O2, O3, and Os) on three architectures (arm, mips, and x86). Dataset-1 consists of 913,508 functions, which are further divided into three subsets (XO, XC, and XA) to fulfill different tasks. We also use 10-fold cross-validation to split Dataset-2 into three disjoint subsets of functions for training, validation, and testing, respectively.

Dataset-2 is designed specifically for evaluating IoTSim’s capability in vulnerability detection. This dataset builds upon embedded firmware and vulnerabilities in previous works [50,63], including 20 firmware images from three vendors (D-Link, TP-Link, and NetGear) and 48 vulnerable functions from the OpenSSL project, as shown in Table 1.

#### 5.1.3. Metrics

The performance of IoTSim and the baselines is measured using the following metrics:

- Recall represents the ratio of correctly matched functions to the total number of function pairs with similar functions. A high recall suggests a low false-negative rate;
- Precision denotes the ratio of correctly matched functions to the total number of function pairs predicted as similar. High precision indicates a low false-positive rate;
- MRR stands for Mean Reciprocal Rank, which is a relative score that calculates the average or mean of the inverse of the ranks at which the first relevant function is retrieved for a set of queries.

### 5.2. Evaluation on Multiple Scenarios

In this subsection, we conduct experiments to evaluate the performance of IoTSim and other baselines at the function level. All evaluations in this subsection are conducted on Dataset-1. We perform IoTSim and baselines under multiple scenarios, including cross-architecture, cross-compiler, and cross-optimization scenarios, to thoroughly assess their performance. In a real-world BCSD task at the function level, a queried function is typically compared to numerous candidate functions to calculate similarity and retrieve the optimal result. Hence, we employ multiple pool sizes to evaluate IoTSim’s performance in different scenarios and discuss the effects of pool sizes on BCSD approaches. Furthermore, to evaluate the contribution of Data-based Control Flow Graphs (DCFGs) to IoTSim, we configure IoTSim${}_{CFG}$ using Control Flow Graphs (CFGs) instead of DCFGs.

Table 2, Table 3 and Table 4 report the recall@1 and MRR results for each approach across different compilers (XC), optimization levels (XO), architectures (XA), and combined scenarios (i.e., XO + XC, XO + XA and All) in different pool sizes (10, 100 and 1000). IoTSim consistently outperforms all the baselines in terms of average recall@1 and MRR by considerable margins.

For example, in the XC experiment with a pool size of 10, IoTSim achieves a recall@1 of 0.981 and a MRR of 0.970, which represents improvements of 4.6%, 13.2%, and 4.5% over SAFE, PalmTree, and Trex, respectively. Since IoT firmware originates from diverse architectures in the real world, we also evaluate IoTSim and other baselines in the cross-architecture scenario. In the XA experiment with a pool size of 100, IoTSim surpasses its closest competitor baseline (Trex [13]) by 0.426 for the recall@1, and over 50% for the MRR. We observe that SAFE achieves only a recall@1 of 0.014, while PalmTree fails in the cross-architecture scenario. PalmTree specifically focuses on the x86 mono-instruction set and is unable to handle functions from different architectures. Although SAFE trains its model on different instruction sets, it remains challenging to establish semantic relations between instructions from diverse architectures and embed similar functions from different architectures into comparable embeddings. This limitation is also acknowledged in the Github issues (https://github.com/gadiluna/SAFE/issues/4, accessed on 30 March 2023), which shows current SAFE hardly supports cross-architecture BCSD tasks.

IoTSim addresses the challenge of different architectures by converting binary code into microcode. To tackle out-of-vocabulary (OOV) issues, IoTSim substitutes OOV words with their base tokens, preserving their semantics. This allows IoTSim to capture the basic semantics of OOV words, alleviating the problem of lost semantics due to OOV issues.

**Figure 8:**
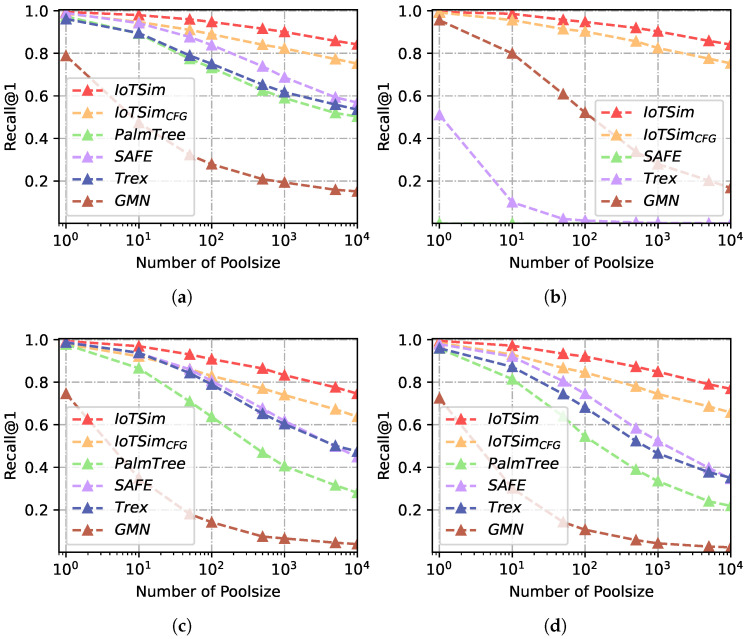

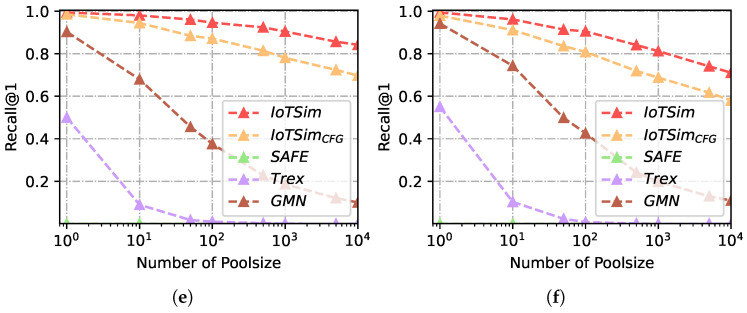
Recall@1 results of multiple scenarios with different poolsizes. (**a**) Recall@1, XO. (**b**) Recall@1, XA. (**c**) Recall@1, XC. (**d**) Recall@1, XO + XC. (**e**) Recall@1, XO + XA. (**f**) Recall@1, XA + XC.

**Figure 9:**
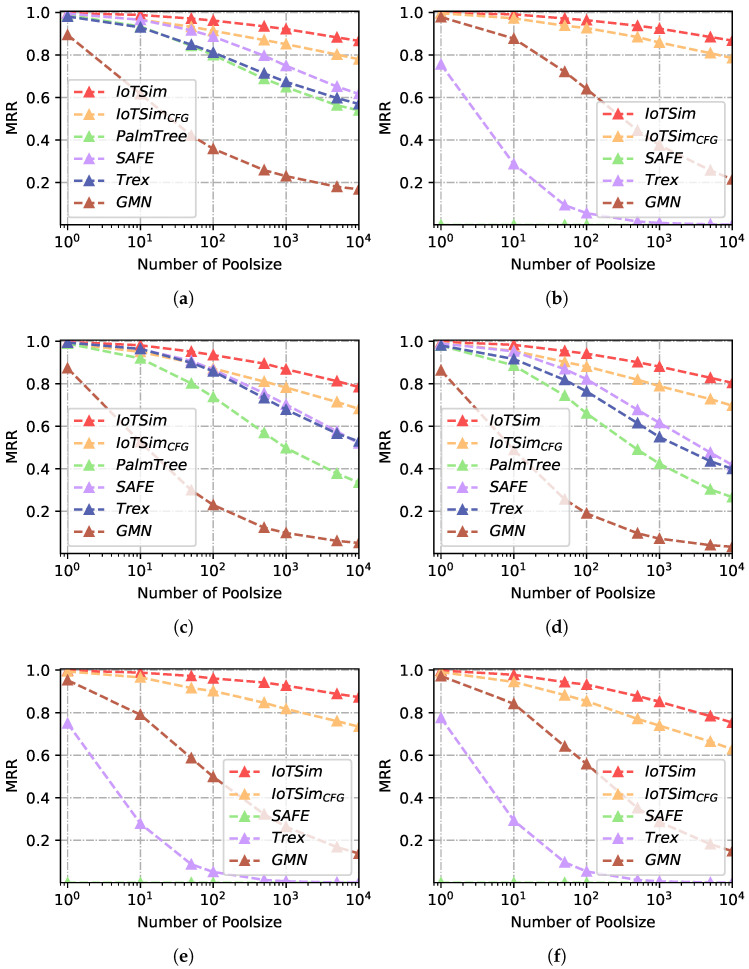
MRR results of multiple scenarios with different poolsizes. (**a**) MRR, XO. (**b**) MRR, XA. (**c**) MRR, XC. (**d**) MRR, XO + XC. (**e**) MRR, XO + XA. (**f**) MRR, XA + XC.

The experimental results, presented in Table 2, Table 3 and Table 4, indicate a decline in recall@1 and MRR for BCSD approaches as the pool size increases. In order to further investigate the impact of the pool size on the performance of BCSD approaches, we conduct experiments with pool sizes ranging from $10^{0}$ to $10^{4}$. Figure 8 and Figure 9 illustrate the results of these experiments with a variety of pool sizes (1, 10, 50, 100, 500, 1000, 5000, and 10,000) under various experimental settings. For the sake of observation, we use a logarithmic x-axis in Figure 8 and Figure 9. As the pool size increases, the performance of all BCSD approaches decreases. Compared to IoTSim, all baselines’ relative performance worsens as the pool size increases. IoTSim does not display sharp drops in its performance, while the baselines’ performance generally declines more rapidly once poolsize over $10^{2}$. For example, when the pool size is $10^{0}$, the recall@1 achieved by SAFE and IoTSim is 0.989 and 0.994, respectively, in the XO experiments. When the pool size is $10^{4}$, IoTSim achieves a recall@1 of 0.842 (−15.3%), demonstrating greater stability compared to other baselines. In contrast, SAFE only achieves a recall@1 of 0.569 (−42.5%) when the pool size is $10^{4}$. This suggests that our approach is not affected by the pool size as much as other baselines.

### 5.3. Ablation Study

We conduct experiments on IoTSim to evaluate the contributions of our proposed block relation matrix.

In binary functions, the relations between basic blocks are various. Previous approaches use control flow graphs as function structure features, which mainly consider connected relations without data dependence, edge direction, and address adjacency. We propose DCFGs to capture functions’ structures deeply and generate block relation matrix integrating data dependence, directed adjacency, and address adjacency. In Table 2, Table 3 and Table 4, IoTSim outperforms IoTSim${}_{CFG}$ in terms of average recall@1 and MRR by considerable margins. Figure 8 and Figure 9 also show IoTSim achieves higher performance than IoTSim${}_{CFG}$ and is less impacted by poolsize than IoTSim${}_{CFG}$. For example, IoTSim improves the recall@1 by 21.6% over IoTSim${}_{CFG}$ in the All scenario (poolsize = $10^{3}$). With the help of the DCFGs, IoTSim captures more accurate data and control flow structures of functions, which makes IoTSim identify similar functions and distinguish dissimilar functions with a higher recall@1 and MRR.

### 5.4. Applications

We evaluate IoTSim and baselines in two practical applications: vulnerability detection and component analysis.

#### 5.4.1. Vulnerability Detection

Vulnerability detection is a crucial application in the field of computer security. Within the IoT context, routers play a vital role in facilitating communication between connected IoT devices. In this subsection, we gather 20 firmware images of routers from three vendors, namely D-Link, TP-Link, and NetGear. We identify five known vulnerabilities in OpenSSL from the CVE database. These firmware images and vulnerabilities are then used to evaluate the performance of IoTSim and other baselines in the vulnerability detection tasks. In total, there are 47,090 functions, including 48 related vulnerable functions. In order to construct the vulnerability repository, we utilize IoTSim to generate function embeddings for each vulnerable and patched functions. During the vulnerability detection phase, we use all functions in the firmware libraries as function queries and search for the most similar function in the built vulnerability repository.

**Figure 10:**
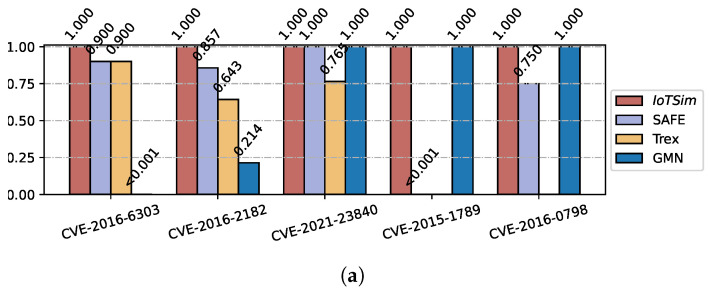

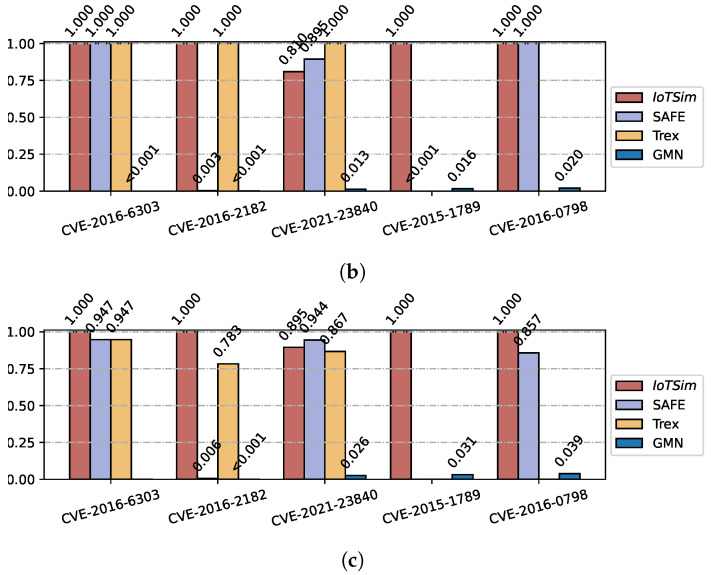
Results of real-world vulnerability detection. (**a**) Recall. (**b**) Precision. (**c**) F1-score.

Figure 10 shows the results of the recall, precision, and F1-score of each CVE vulnerability. We compare IoTSim with other baselines. It is clear that for most of the CVEs, IoTSim’s performance is significantly higher than the state-of-the-art approaches, e.g., SAFE and Trex. For instance, in the case of CVE-2016-2182 from the OpenSSL project, our method achieves a recall of 100%, successfully identifying all 14 vulnerable functions. In contrast, SAFE and Trex achieve recall values of 85.7% and 64.3%, respectively. Furthermore, it is worth noting that SAFE and Trex fail to obtain any recall for CVE-2015-1789 due to their reliance on capstone and objdump, which cannot deal with complex binary formats. For example, objdump (version 2.34) cannot extract function features from ELF files without a section table, which makes them fail to perform BCSD task in these binaries. This reflects the difficulty of the BCSD task in IoT scenarios, and the necessity of our approach. The results demonstrate that IoTSim can be effectively deployed as a reliable tool for detecting vulnerabilities in IoT scenarios.

#### 5.4.2. Component Analysis

BCSD approaches can provide component analysis for unknown executable binary files and match symbol tables and source code for reference, making it easier for reverse engineers to analyze unknown binaries in IoT security. In this section, we use the OpenSSL project, which is widely used in IoT firmware, as the benchmark to evaluate the performance of our proposed approach and other baselines on component analysis. Given an input binary file, the BCSD approaches compare it with our labeled binaries that contain debug information to match symbol tables.

**Figure 11:**
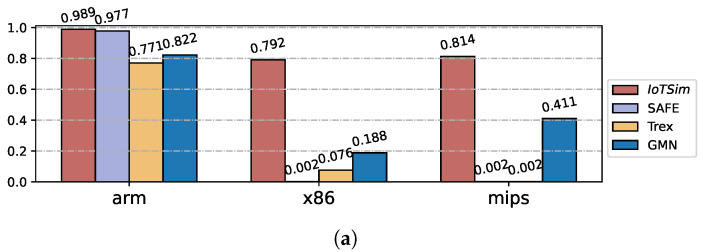

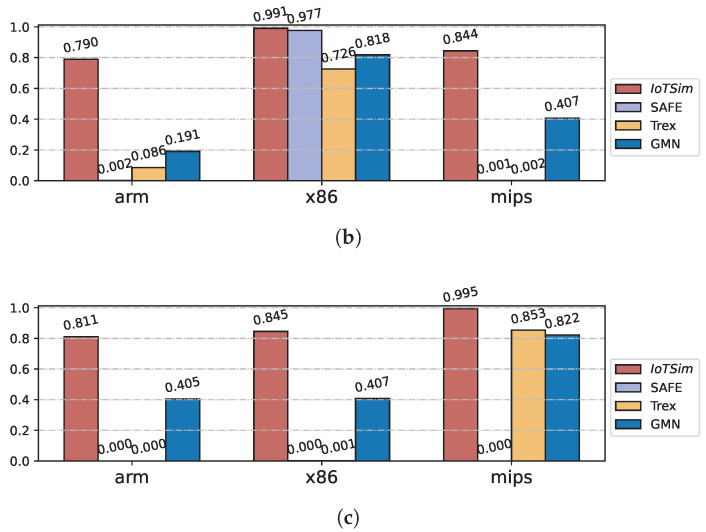
Results of the component analysis on different architectures. (**a**) arm. (**b**) x86. (**c**) mips.

Figure 11 shows recall results in component analysis with labeled binaries from different architectures (i.e., x86, arm, and mips). IoTSim achieves high recalls in all component analysis experiments. For example, IoTSim obtains a recall of 0.814 when the input files are from mips and the labeled binaries are from arm, which means more than 80% functions in the input binaries can be correctly matched to their symbol tables and their source code. This significantly reduces the manual burden when analyzing unknown binary files. Compared with the state-of-the-art approaches, IoTSim achieves the average recall of 0.874, which improves the recall by 3.0×, 0.8× and 2.1×, compared to SAFE, GMN, and Trex.

## 6. Conclusions

In this paper, we propose a novel IoT-oriented binary code similarity detection approach, called IoTSim. Our approach leverages a customized transformer-based language model with disentangled attention to generate embeddings for basic blocks. To address OOV challenges, we introduce a pre-training task called BTP that captures basic semantics for unseen tokens. To help IoTSim understand multiple relations between basic blocks, we integrate directed jumps, data dependency, and address adjacency to build block relation matrix. We then assign different weights to different relations in block relation matrix and use multi-layer GCN to generate function embeddings.

We implemented a prototype of IoTSim and conducted experiments to evaluate its performance. The experiment results show that IoTSim surpasses the state-of-the-art approaches Trex, SAFE, and PalmTree. Additionally, we observe that data-based control flow graphs have positive effects for IoTSim. In real-world applications, IoTSim proves valuable in helping researchers detect vulnerabilities and identify components in unknown binaries within various IoT firmware. These findings demonstrate that our proposed BCSD approach contributes to practical applications in security analysis within the IoT ecosystem, relieving researchers from the burdensome task of security analysis.

## Figures and Tables

**Table 1:** The vulnerabilities involved in Dataset-2.

CVE	Vulnerable Function	Confirmed #
CVE-2016-6303	MDC2_Update	10
CVE-2016-2182	BN_bn2dec	14
CVE-2021-23840	EVP_DecryptUpdate	17
CVE-2015-1789	X509_cmp_time	3
CVE-2016-0798	SRP_VBASE_get_by_usr	4

**Table 2:** BCSD results on multiple scenarios at the function level (poolsize = 10).

	Recall@1							MRR						
**Models**	**XO**	**XA**	**XC**	**XO + XC**	**XO + XA**	**XA + XC**	**All**	**XO**	**XA**	**XC**	**XO + XC**	**XO + XA**	**XA + XC**	**All**
SAFE	0.942	0.100	0.937	0.920	0.090	0.103	0.085	0.965	0.287	0.963	0.952	0.278	0.293	0.278
PalmTree	0.892	-	0.866	0.814	-	-	-	0.934	-	0.919	0.886	-	-	-
GMN	0.473	0.516	0.349	0.301	0.333	0.374	0.296	0.616	0.655	0.525	0.488	0.506	0.542	0.479
Trex	0.895	0.800	0.938	0.872	0.680	0.744	0.624	0.930	0.877	0.964	0.916	0.791	0.842	0.753
IoTSim	0.980	0.986	0.969	0.972	0.980	0.962	0.963	0.988	0.991	0.981	0.983	0.987	0.977	0.978
IoTSim${}_{CFG}$	0.948	0.956	0.922	0.930	0.945	0.912	0.899	0.965	0.972	0.950	0.954	0.965	0.944	0.936

**Table 3:** BCSD results on multiple scenarios at the function level (poolsize = $10^{2}$).

	Recall@1							MRR						
Models	**XO**	**XA**	**XC**	**XO + XC**	**XO + XA**	**XA + XC**	**All**	**XO**	**XA**	**XC**	**XO + XC**	**XO + XA**	**XA + XC**	**All**
SAFE	0.839	0.014	0.806	0.745	0.010	0.008	0.006	0.886	0.056	0.866	0.821	0.051	0.053	0.050
PalmTree	0.732	-	0.638	0.545	-	-	-	0.800	-	0.738	0.660	-	-	-
GMN	0.279	0.319	0.142	0.106	0.132	0.164	0.113	0.359	0.405	0.230	0.190	0.218	0.250	0.197
Trex	0.750	0.521	0.790	0.681	0.376	0.426	0.316	0.811	0.639	0.859	0.764	0.499	0.559	0.448
IoTSim	0.948	0.947	0.909	0.921	0.946	0.906	0.912	0.962	0.963	0.935	0.941	0.960	0.931	0.936
IoTSim${}_{CFG}$	0.890	0.903	0.830	0.846	0.871	0.808	0.803	0.913	0.926	0.872	0.881	0.901	0.854	0.848

**Table 4:** BCSD results on multiple scenarios at the function level (Poolsize = $10^{3}$).

	Recall@1							MRR						
**Models**	**XO**	**XA**	**XC**	**XO + XC**	**XO + XA**	**XA + XC**	**All**	**XO**	**XA**	**XC**	**XO + XC**	**XO + XA**	**XA + XC**	**All**
SAFE	0.687	0.002	0.618	0.523	0.001	0.001	0.001	0.749	0.010	0.702	0.616	0.007	0.007	0.007
PalmTree	0.590	-	0.406	0.335	-	-	-	0.648	-	0.497	0.424	-	-	-
GMN	0.193	0.190	0.066	0.042	0.053	0.073	0.038	0.230	0.237	0.098	0.071	0.085	0.112	0.070
Trex	0.627	0.280	0.603	0.465	0.186	0.198	0.133	0.673	0.375	0.678	0.549	0.263	0.286	0.208
IoTSim	0.901	0.903	0.833	0.849	0.905	0.812	0.832	0.922	0.924	0.868	0.880	0.926	0.851	0.867
IoTSim${}_{CFG}$	0.823	0.825	0.740	0.744	0.781	0.688	0.684	0.851	0.858	0.782	0.789	0.818	0.739	0.734

## Data Availability

Available upon request.

## Abbreviations

The following abbreviations are used in this manuscript:

BCSD	Binary Code Similarity Detection
IoT	Internet of Things
OOV	Out-of-Vocabulary
TPC	Third-Parity Components
ISA	Instruction Set Architecture
CVE	Common Vulnerabilities and Exposures
ML	Machine Learning
CFG	Control Flow Graph
IR	Intermediate Representation
GNN	Graph Neural Network
NLP	Natural Language processing
GCN	Graph Convolutional Network
